# The invisible minority: A call to address the persistent socioeconomic diversity gap in U.S. medical schools and the physician workforce

**DOI:** 10.3389/fpubh.2022.924746

**Published:** 2022-07-29

**Authors:** Kevin E. Salinas, Hillary Brenda Nguyen, Sophia C. Kamran

**Affiliations:** ^1^Harvard Medical School, Boston, MA, United States; ^2^Department of Radiation Oncology, Massachusetts General Hospital, Boston, MA, United States

**Keywords:** diversity & inclusion, physician workforce, socioeconomic diversity, first generation, low income, medical school

In recent years, there has been substantial national discourse surrounding diversity in healthcare and its impact on both trainees and patients. Diversity at the faculty level can foster a more welcoming training environment, diverse teams can prepare students from majority groups to care for diverse populations, and a diverse team of providers can more thoroughly mitigate the impact of implicit biases on patient care (1). The issue of diversity is particularly pressing in healthcare given the intimate nature of clinical encounters and the trust required to appropriately serve an increasingly heterogenous patient population. This priority of building a workforce representative of all our patients demands further investigation into how current workforce diversity trends in healthcare reflect the economic and educational diversity of the United States (U.S.) given that these factors are largely intersectional and include individuals from a wide array of backgrounds not easily captured in affinity groups that recognize individuals' experiences based on ethnicity, race, gender identity, or sexual orientation.

## Widening U.S. socioeconomic gap

Data trending income from the 1970s through the 2010s suggest that, after adjusting for inflation, the wealthiest Americans have had increasing incomes while the poor have had constant or even decreased incomes. This degree of income inequality has not been seen in the U.S. since the Great Depression (2). Similarly, while more of the U.S. population has enrolled in and completed some level of postsecondary education since the turn of the century, historical disparities in educational attainment remain high, with Black and Hispanic adults exhibiting lower levels of educational attainment compared to White adults (3). The impact of income and educational inequality on health is evident as low-income jobs can present barriers to obtaining insurance, which themselves result in barriers to receiving adequate care. Similarly, reduced educational attainment is correlated with decreased health literacy, directly impacting chronic disease perception and management (4–6). Given the role of systemic racism and oppression in the U.S., race, ethnicity, socioeconomic status, and educational background are inevitably intertwined—individuals with higher levels of educational attainment are more likely to be White and employed, have higher salaries, and own homes (6–9). The impacts of these systemic challenges have been particularly hard-felt during the COVID-19 pandemic, as socially and economically marginalized groups were disproportionately impacted in terms of morbidity and mortality, often due to broader systemic disinvestment in their communities (10–13).

## First-generation, low-income

Despite the well-investigated and well-understood impacts of educational attainment and income on health, efforts to promote diversity in healthcare have, until recently, primarily centered on areas of identity such as race or ethnicity, gender, and sexual orientation. Still, these areas fail to specifically foster community and promote resource allocation for the often intersectional first-generation/low-income (FGLI) community. This identity, shaped by the lived experiences of those who are first-generation college students (and therefore, first-generation medical students) and/or low-income, is combined into a single label because household income is a powerful predictor of parental educational attainment. There is little known about this entity in medicine. An *Analysis in Brief* report by the Association of American Medical Colleges (AAMC) assessing economic diversity of U.S. medical students compared to U.S. Census data from 1988 to 2017 identified that approximately three-quarters of all medical students come from the top two household income quintiles, with approximately half from the top 20% and one-quarter from the top 5% (14). These figures have been somewhat stagnant, with representation of those from the bottom income quintile not surpassing 6% since 1987. Even when de-aggregated by race/ethnicity group—including Asian, Black, Hispanic, and White—top quintile households are disproportionately overrepresented (15). Conversely the bottom 3 income quintiles are consistently underrepresented. Notably, this disparity based on income captures low-income White individuals who may not otherwise have affiliation to traditional affinity groups based on the areas of identity previously mentioned. Thus, highlighting the FGLI community as a way to improve awareness and representation of underrepresented low-income groups will be key not only in advancing efforts to reduce health inequity based on income status, but also in providing community to low-income White individuals who may otherwise not be identified as forming part of an underrepresented identity in healthcare.

## Untapped potential

The authors of this piece are all FGLI, and using our own backgrounds growing up in families without higher education, we have a deep understanding that—without being steeped in medicine and with low baseline health literacy—to many patients a simple doctor's visit can feel intimidating, unwelcoming, and confusing. One author is a first-generation Mexican-American from the Texas US-Mexico border and son of an HVAC technician and a homemaker, another author is the child of Vietnamese refugees and the first in their family to attend higher education beyond high school, and the final author is a third-generation Mexican-American from the Midwest, and the first in her family to attend higher education beyond high school. We believe that the lack of representation and visibility of FGLI trainees in healthcare reduces their ability to contribute positively to the healthcare field overall. Their worldview, when applied to “holistic” and patient-centered care, brings a unique cultural understanding, compassion, and empathy to serving individuals facing insufficient or lack of insurance coverage, difficulty affording medications, or challenging socioeconomic circumstances. FGLI trainees and physicians are also uniquely poised to develop creative solutions for patients by using their own understanding of navigating situations with limited resources. As current FGLI healthcare providers engaging with patients, in our experience, we have consistently received positive feedback on our ability to thoroughly explain medical jargon, demystify treatment algorithms, and give patients more agency when making healthcare decisions. Our backgrounds and experiences navigating the medical system with our relatives with limited educational background and health literacy have inherently positioned us to better communicate with patients across the wide economic and educational spectrum of the U.S. population. In light of the vaccine hesitancy and other disparities exacerbated by the COVID-19 crisis, FGLI physicians have the potential to be leaders in addressing health issues at individual and systemic levels.

It is important to note that the FGLI experience differs from a traditional underrepresented racial/ethnic minority experience in that, as demonstrated by previously mentioned data on income backgrounds of matriculating students, those from underrepresented race/ethnicity groups who come from families in higher income brackets are disproportionately represented in entering medical school classes. Thus, the majority of medical students from underrepresented backgrounds based on race/ethnicity do not experience a disadvantage in terms of access to financial, social, and academic resources in the same way as FGLI students do. Moreover, in many cases, students who are FGLI are of double or triple minority status, as their FGLI identity intersects with other URM identities based on race/ethnicity, gender identity, or sexual orientation.

In the authors' personal experiences, the culture of medicine and medical training can at times result in an alienating experience. From expectations of professionalism based on standards derived by the historically dominant group—upper class White male physicians—to an inability to relate to the experiences of faculty and their peers who were raised in physician or high-income families, the FGLI experience in medical training can oftentimes result in a lack of belonging. This cultural disconnect can have direct ramifications on the ability to form meaningful mentorships and connections with peers that contribute to academic and long-term career success.

Due to its nascent recognition within healthcare, the FGLI community is sorely lacking visibility, celebration, and representation. Previously existing items on the American Medical College Application Service (AMCAS) allowed for indication of patient educational level, fee assistance program (FAP) waiver status, socioeconomic status based on parental education and occupation, and socioeconomic/childhood educators. These factors indirectly represented a student's FGLI status, however did not use the specific term “first generation” used in part to unify and identify the FGLI community, especially at the college level (16). We applaud steps taken by the AAMC such as introducing a “First Generation College Student Indicator” on the AMCAS in 2017. In addition, the National Institutes of Health (NIH) recognizes that scientific research opportunities are not equally available to all, and thus created a definition specifically for individuals from disadvantaged backgrounds which can be considered for grant applications (17). This is based on data demonstrating that individuals from low socioeconomic backgrounds have minimal representation in biomedical research.

## Steps forward

**Figure 1:**
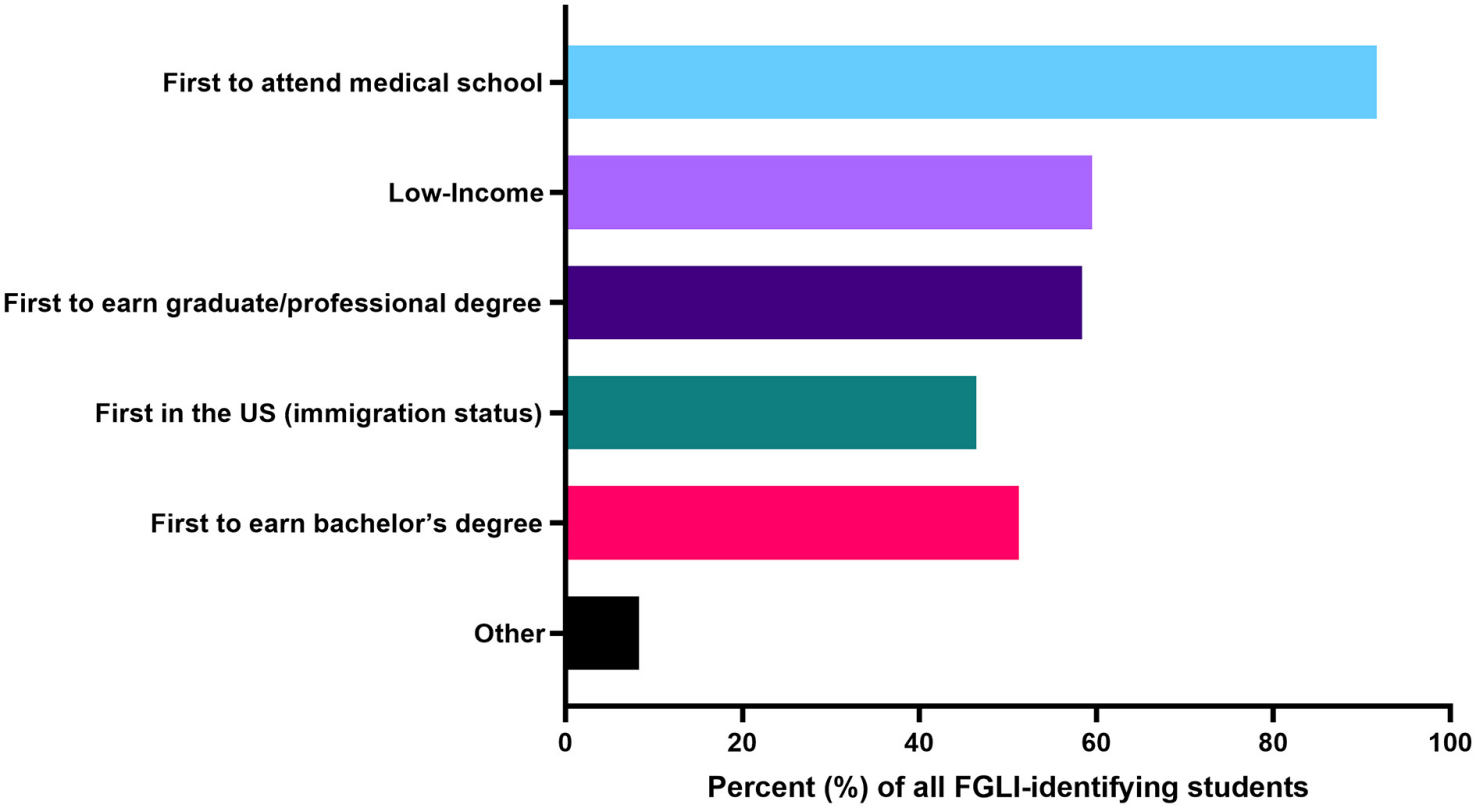
In a survey of all medical at Harvard Medical School in 2020, 84 students identified as first-generation/low-income, with 46.4% of those identifying as being the first generation in their family to be in the US (immigration status), 91.7% identifying as the first in their family to attend medical school, 51.1% identifying as the first in their family to earn a bachelor's degree, 58.3% identifying as the first in their family to earn a graduate/professional degree, and 59.5% identifying as low-income.

Nevertheless significant challenges for the community remain, which include a lack of financial resources and academic skills training, culture shock, and long-term financial concerns including delayed income and student debt. With dedicated individual and institutional backing, several actionable steps can contribute to improving recruitment, retainment, and career support for FGLI trainees and physicians. Firstly, establishing a working institutional definition of FGLI allows for rapid mobilization of concrete, welldefined resources to support students from more under-resourced backgrounds. At Harvard Medical School, a recent survey among all medical students has brought to light the broad experiences within the first-generation community (Figure 1). By seeking more rigorous disaggregated data, we hope to better target disparities in social, cultural, and economic capital.

Recent holistic approaches to addressing issues with diversity in many regards have been suggested. Suggested strategies include mention of diversity enhancement in institutional mission statements, anonymous admissions committee voting, recruiting larger groups of faculty screeners as a way to mitigate individual implicit and explicit biases, requiring and reviewing admission committee member implicit association test scores, blinding academic metrics during application review, and removing photos from application files during application review. These are all steps that can ultimately contribute to recruitment and ultimately foster a feeling of belonging for FGLI students (18). Nevertheless, there are other FGLI-specific approaches for supporting this community in education and training. In order to address financial burden and concerns, support can be demonstrated *via* scholarship and loan forgiveness programs, grocery partnerships, subsidized rent policies, professional clothing drives, textbook libraries, medical equipment donations, prepaid institutional subscriptions to outside study resources, emergency funds, and school-sponsored transportation and social activities. To address sociocultural disconnect and lack of social capital in higher education, facilitating committed formal and informal mentoring opportunities with other students, residents, faculty, and alumni can further contribute to the long-term success of FGLI individuals in healthcare. Beyond the institutional level, focusing recruitment efforts in lower-income and disadvantaged communities, including systemically disadvantaged and historically under-resourced communities such as FGLI immigrant and refugee families, can strengthen the academic pipeline for FGLI students exploring health careers. Providing greater support for prospective students and applicants throughout the admissions process and further publicizing financial support such as the AAMC Fee Assistance Program and Loans for Disadvantaged Students awards can both promote a culture of inclusion and ease financial concerns. Additionally, these efforts to shed light on and celebrate the FGLI identity will allow for gathering of much-needed data on FGLI physicians and trainees to understand their outcomes considering unknown attrition rates at all levels.

## Conclusions

Now more than ever, given the state of income and education inequality in the U.S. and the resulting consequences on health, it is time to act deliberately to change the face of healthcare so that it more appropriately reflects the communities we serve. Though currently underrepresented, the life experiences of FGLI trainees and physicians provide unique and irreplaceable perspectives with which to understand and address structural injustices and disparities in healthcare. With dedicated investment, these insights promise to improve not only the quality of care provided to patients, but also the cultural sensitivity of the entire healthcare system.

## Author contributions

All authors listed have made a substantial, direct, and intellectual contribution to the work and approved it for publication.

## Conflict of interest

The authors declare that the research was conducted in the absence of any commercial or financial relationships that could be construed as a potential conflict of interest.

## Publisher's note

All claims expressed in this article are solely those of the authors and do not necessarily represent those of their affiliated organizations, or those of the publisher, the editors and the reviewers. Any product that may be evaluated in this article, or claim that may be made by its manufacturer, is not guaranteed or endorsed by the publisher.
